# Translation, cultural adaptation and validation of a patient‐reported experience measure for children

**DOI:** 10.1111/hex.13924

**Published:** 2023-12-07

**Authors:** Anna Nordlind, Agneta Anderzén‐Carlsson, Ann‐Sofie Sundqvist, Karin Ängeby, Jo Wray, Geralyn Oldham, Ann‐Charlotte Almblad

**Affiliations:** ^1^ School of Health Sciences, Faculty of Medicine and Health Örebro University Örebro Sweden; ^2^ Department of Paediatric Medicine County Hospital Karlstad Karlstad Sweden; ^3^ University Health Care Research Centre, Faculty of Medicine and Health Örebro University Örebro Sweden; ^4^ Centre for Clinical Research and Education Region Värmland Karlstad Sweden; ^5^ School of Education, Health and Social Studies Dalarna University Falun Sweden; ^6^ Centre for Outcomes and Experience Research in Children's Health, Illness and Disability (ORCHID) Great Ormond Street Hospital for Children NHS Foundation Trust London UK; ^7^ Data Research, Innovation and Virtual Environments (DRIVE) Unit Great Ormond Street Hospital for Children NHS Foundation Trust London UK; ^8^ Department of Women's and Children's Health Uppsala University Uppsala Sweden; ^9^ Children Hospital and Emergency Region Uppsala Uppsala Sweden

**Keywords:** children, cognitive interviews, content validity index, PREM, validation

## Abstract

**Background:**

There is no national, validated, generic patient‐reported experience measure (PREM) for children under 15 years of age in Sweden. A recent cross‐sectional study found no consensus in how children's voices are heard in paediatric health care, as well as a lack of validated questionnaires.

**Aim:**

The aim of this study is to translate, adapt and validate the six versions of the Children's and Young People's PREM for use in a Swedish health care context.

**Design:**

An exploratory sequential mixed‐method design including cognitive interviews and content validity index (CVI) was used. The interviews focused on evaluating children's understanding of the questionnaire, and the CVI was used to further adjust the relevance of the questionnaire.

**Participants:**

A convenience sample of 62 children participated in the cognitive interviews and an additional convenience sample of 42 children was included in the CVI testing. The children, aged 8–16 years, were attending routine visits at paediatric departments in a county hospital and a children's hospital in the mid‐Sweden region between October 2020 and June 2022.

**Results:**

The translation, adaptation and validation process identified several issues regarding the understanding of the questionnaire in a Swedish context. Adaptations were made based on issues related to context, wording and the structure of the questions. CVI testing resulted in the removal of 3–10 questions in each of the different versions of the questionnaire.

**Conclusion:**

The study has resulted in six face‐ and content‐validated Swedish versions of the questionnaire ready for pilot testing. Although the versions of the original questionnaire were developed in collaboration with children in the United Kingdom, this did not mean that they could automatically be used in a Swedish health care context. This study confirms the importance of a rigorous process of adaptation and validation to ensure quality and applicability to children accessing health care in different countries.

**Patient or Public Contribution:**

Children's views have guided the development of the original instrument and its adaptation to the Swedish health care context. Due to the strong patient involvement in the process of developing the Swedish versions of the questionnaire, the research group made a pragmatic decision to have no other patient contribution in the study.

## 1 INTRODUCTION

Patient‐reported experience measures (PREMs) are questionnaires measuring patients' experiences of receiving health care, and include assessment of experiences, perceptions and satisfaction with care.^1^ There is no national, validated, generic PREM for Swedish children under 15 years of age.^2^ A recent cross‐sectional study showed no consensus in how children's voices are heard in Swedish paediatric departments. Fewer than half of the participating departments reported using questionnaires completed by children themselves and few used validated questionnaires,^3^ indicating that children's rights in health care, article 12 of the United Nations Convention on the Rights of the Child (UNCRC) and Swedish laws^4^, ^5^, ^6^ are not being fully implemented. In this article, we chose to use the term children for all children and adolescents up to the age of 17 years, in line with the definition of a child in the Convention on the Rights of the Child.^6^

## 2 BACKGROUND

Several PREMs for children exist, most adapted for special patient groups. However, how these questionnaires were developed and what issues they include vary.^7^, ^8^, ^9^, ^10^ It can be challenging for adults to take children's views into account; adult descriptions of children's experiences may differ from the experience described by children themselves.^11^, ^12^ It is, therefore essential to collaborate directly with children to determine which aspects of care are important to them, as adults cannot fully act as a proxy for children. This was a key consideration in the development of a generic Children's and Young People's Patient‐Reported Experience Measure (CYP‐PREM), which was developed in collaboration with children aged 8–16 years who had experienced hospital inpatient and/or outpatient care at a children's hospital in the United Kingdom.^13^ The questionnaire is available in six versions: both for inpatient and outpatient settings and for three age groups (8–11, 12–13 and 14–16 years), respectively. It covers three themes: hospital facilities, hospital staff and treatment and tests. An additional section focuses on supplementary questions. Children were also involved in the graphic design of the questionnaire. The graphic designs of the versions for children aged 8–11 and 12–13 years have an animal design and texts about animals, and the layout for the older group has a cartoon design (see the Supporting Information Materials for examples). In a Danish study which translated and tested the CYP‐PREMs in an outpatient setting, most children found the questions easy to read and understand. The children were positive about giving voice to their experiences and the layout of the questionnaire contributed to this.^14^

As the development of the original CYP‐PREM aligns with the UNCRC and since patients' views on care are important for quality improvement,^2^, ^15^ the six versions of the CYP‐PREM were chosen for adaptation to a Swedish health care context.

## 3 AIM

The aim of this study was to translate, adapt and validate the six versions of the CYP‐PREM for use in a Swedish health care context.

## 4 METHODS

### 4.1 Design

An exploratory sequential mixed‐method design^16^ was used. Translation and cultural adaptation of the questionnaire followed the recommendations of the International Society for Pharmacoeconomics and Outcomes Research,^17^ presented below. This validation process clarified the need to proceed with use of the content validity index (CVI),^18^, ^19^ a procedure in line with an exploratory sequential mixed‐method design.^16^

#### 4.1.1 Phase 1: Translation and cultural adaptation

The researchers involved in the development of the original CYP‐PREM granted permission to translate and adapt the questionnaire. The research groups in Sweden and the United Kingdom agreed on the process for translation and cultural adaptation of the questionnaire into Swedish. The groups include members with extensive experience in paediatric care, as well as previous experience in instrument development, including the current method of translation, adaptation and validation and experience with CVI testing. Two researchers (A. N. and A‐C. A.) independently translated the six versions of CYP‐PREM from English into Swedish. The Swedish research group (A. N., A. A‐C., A‐S. S., K. Ä. and A‐C. A.) discussed necessary linguistic and cultural adjustments, as well as adjustments to achieve a more homogeneous language throughout all versions of the questionnaire until consensus was reached. A professional translator conducted a conceptual translation of the questions and response options, capturing the meaning of the content, rather than a word‐by‐word translation.

The original questions, translation and back‐translated version of the questions were included in a matrix to facilitate comparison between the different versions and to assess the translation. A bilingual (Swedish–English) clinician assisted the English‐speaking researchers (J. W. and G. O.) in comparing the Swedish versions of the questionnaire with the original, to ensure semantic alignment between the Swedish and English versions. Differences in opinions about questions and response options were discussed until consensus was reached between all the researchers. Remaining inconsistences in language, such as spelling and similar errors, were identified and corrected. Preliminary Swedish versions of CYP‐PREM were developed, using the same graphic design as in the original versions. The researchers were responsible for the development and a person knowledgeable in graphic design helped with the practical adaptation.

Two researchers (A. N. and A‐C. A.) conducted cognitive interviews^20^, ^21^ between October 2020 and January 2022 in the paediatric departments of a county hospital and a children's hospital in the mid‐Sweden region. The children's hospital has 36 inpatient beds in acute, oncology, neurology, urology, orthopaedics and surgery care. There are also 12 day‐care beds, 18 specialist outpatient clinics, a paediatric intensive care unit and a neonatal ward. The county hospital has about 16 inpatient beds caring for all patient groups, a neonatal ward, a day‐care ward with six beds and a specialist outpatient clinic. Patients, in Swedish paediatric care, are usually 0–17 years of age. Children were recruited during an inpatient stay or an outpatient visit (Table 1). As the original CYP‐PREM was intended for the age group 8–16 years, children were eligible for inclusion if they were aged 8–16 years, could understand Swedish and were judged by health care staff as being able to answer the questionnaire and participate in a cognitive interview.

**Table 1:** Demographics of the participants in the cognitive interviews.

Participants^a^	First round (*n* = 18)		Second round (*n* = 19)		Third round (*n* = 17)		Fourth round (*n* = 8)		Total number of participants (*n* = 62)	
	Inpatient (*n* = 10)	Outpatient (*n* = 8)	Inpatient (*n* = 7)	Outpatient (*n* = 12)	Inpatient (*n* = 6)	Outpatient (*n* = 11)	Inpatient (*n* = 3)	Outpatient (*n* = 5)	Inpatient (*n* = 26)	Outpatient (*n* = 36)
Age 8–11	4	4	2	5	1	4	1	2	8	15
Age 12–13	2	1	2	4	1	4	2	1	7	10
Age 14–16	4	3	3	3	4	3	–	2	11	11
Boys	6	2	2	2	1	6	2	3	11	13
Girls	4	6	5	10	4	5	1	2	14	23
Own description of gender	–	–	–	–	–	–	–	–	–	–
Prefer not to answer regarding own gender	–	–	–	–	1	–	–	–	1	–
Other first language^b^	3	–	2	3	–	4	1	–	6	7
First visit	6	1	3	2	3	2	2	–	14	5

^a^Visiting the health care unit regarding, for example, acute infections, blood or tumour disease, heart disease, intestinal disease, metabolic disease, orthopaedic surgery, rheumatism, trauma, urological conditions.

^b^ Albanian, Arabic, Bosnian, English, Hindi, Polish, Spanish.

The Swedish versions of the questionnaire were tested through cognitive interviews to understand the questions from a child's perspective and to identify any problems with the questions, response options or layout. Children were interviewed while completing the age‐appropriate CYP‐PREM. Most children answered all questions. A few children did not complete the questionnaire because they found it too long, did not have enough time or skipped certain questions that they could not answer, as the visit had not finished. They were asked to read the questions out loud and encouraged to ‘think aloud’ to describe their thoughts as they answered the questions. Some children preferred the researcher or parent/carer to read the questions to them. Children and parents/carers were informed that it was the child's view that was important in the study. If the adult was responding on behalf of the child, the researcher actively turned to the child to get the child's point of view. It was judged that there were no obvious differences in the results of the cognitive interviews when the child read the questions themselves compared with when a parent/carer read the questions to the child. Interviews followed a predesigned structured interview guide based on the questionnaire and were audio‐recorded. The interviews focused on hearing children's words to evaluate their understanding of the questions. Children were also asked to define the meaning of selected words and to explain their answers to the questions, so the researcher could judge whether the child understood the questions as intended. All uncertainties that the children highlighted, as well as difficulties identified by the researchers (A. N. and A‐C. A.), (e.g., when the child asked for help, struggled with words or answered questions incorrectly), were fed into a case‐specific matrix for each child, while listening to the recorded interviews.^20^, ^22^, ^23^

##### Analysis of the cognitive interviews

Data from the interviews were analysed by a cross‐case analysis after each round,^22^ where problems were compiled in a cross‐case matrix for each version of the questionnaire. These matrices were summarised by the two researchers who conducted the cognitive interviews, and identified problems and proposals for solutions were discussed within the research group. Together, the Swedish and UK researchers reformulated questions and response options and another round of cognitive interviews was carried out. If, for example, only the youngest children had difficulties understanding the meaning of a specific word, it was consistently amended throughout all versions of the questionnaire. This was a conscious choice aiming to make questions as simple as possible to understand. This iterative process continued until the questionnaire was judged understandable and adapted to a Swedish context, over four rounds.

When summarising the entire process, problems were coded: 1 = *Not relevant or difficulty understanding due to context*, 2 = *Difficulty understanding the meaning of the words*, 3 = *Difficulties in understanding the question's structure or layout*, 4 = *Inconsistent terminology*. Research group discussions ensured that there was consistency in assessment of the individual problems for each question.

The translation, adaptation and validation process ended with proof‐reading of the six versions of the questionnaire to ensure that minor errors were corrected in the templates, and all final translation and adaptation decisions were summarised.

#### 4.1.2 Phase II: CVI)

The next step of the cultural adaptations was to compute CVI scores to test the relevance of the questions and possibly reduce questions in CYP‐PREM based on the children's views. Two researchers (A. N. and A‐C. A.) conducted CVI testing in May–June 2022 in the same paediatric departments as the cognitive interviews. As a minimum of six experts has been suggested, based on a literature review,^19^ the aim was to include seven experts (in this study, children) to validate each of the six versions of the questionnaire. Children were recruited during an inpatient stay or an outpatient visit at different units. They were eligible for inclusion if they were aged 8–16 years, could understand Swedish and were judged by health care staff as being able to answer the questionnaire and rate the relevance of the items. A convenience sample of seven children in each group, in total 42 children (23 girls and 19 boys), was included (Table 2). The participants received verbal information from the researcher and were given an envelope with written information about how to rate the relevance of the questions from the age‐appropriate CYP‐PREM, including image support, a consent form and materials for the CVI. The information to parents/carers was that ‘The child can ask for help from an adult, but it is important that it is the child's views that are expressed’.

**Table 2:** Demographics of the participants in the CVI.

	Inpatient 8–11	Inpatient 12–13	Inpatient 14–16	Outpatient 8–11	Outpatient 12–13	Outpatient 14–16	Total
Boys	1	4	2	4	4	4	19
Girls	6	3	5	3	3	3	23

Abbreviation: CVI, content validity index.

The materials for the CVI included a stack of paper sheets, with each question from the age‐appropriate CYP‐PREM on a separate sheet, and four envelopes marked ‘very important’, ‘important’, ‘not so important’ and ‘not at all important’. The participating children were asked to read each question, rate how important the question was for children to voice their experience of a health care visit and to place the question‐sheet in the most appropriate envelope. The children could choose whether to conduct the CVI at the health care visit or at home. The procedure was carried out without the presence of anyone in the research group, with the exception of one participant, whose first language was not Swedish, who wanted support to ensure that the information was understood correctly.

The data analysis started by entering the results of the collected data into a table, where the participants' ratings of the questions were indicated in numbers: ‘very important’ = 4, ‘important’ = 3, ‘not so important’ = 2, ‘not at all important’ = 1. The higher ratings (3 and 4) received the value 1 and the lower ratings (1 and 2) received the value 0. Item‐CVI (I‐CVI) was calculated to assess the relevance of each individual question by summing the number of 1s and dividing by the number of participants who rated the question. Based on the I‐CVI, the Swedish researchers discussed together with the two researchers from the United Kingdom which questions should be excluded from the different versions of the questionnaire. Agreement was made to remove questions graded ‘not so important’ or ‘not at all important’ by three or more of the seven participating children, as recommended when including 6–10 experts.^18^, ^24^

The overall relevance of the questionnaire was assessed through average scale‐CVI (S‐CVI/Ave), which was calculated by summing the I‐CVI for each question and dividing by the number of questions. An S‐CVI/Ave of 0.90 or higher is recommended for a scale to be judged as having excellent content validity; a minimum S‐CVI/Ave of 0.80 can be tolerated.^18^

### 4.2 Ethical considerations

Both phases of the study were approved by the Swedish Ethical Review Authority (Dnr 2019‐01203, 2020‐02350, 2022‐00837‐02) and adhered to the principles embodied in the Declaration of Helsinki.^25^ Children in health care are a vulnerable group in a dependent position. It is the researchers' responsibility to provide children with adapted information about the voluntariness of participation and the right to terminate at any time.^26^ Children were informed about the study using age‐appropriate verbal and written information. The researchers were not involved in the care of the participants in the cognitive interviews. However, they were involved in the care of some children enroled in the CVI testing, but this was not considered as an ethical issue since the children were not asked to answer any questions concerning their actual care. In both phases of the study, the voluntariness of participating was made clear. Written assent for participation was obtained from the child and written informed consent from a parent/carer was obtained if the child was younger than 15 years of age. Children older than 15 years of age provided written informed consent.

## 5 FINDINGS

### 5.1 Translation, cultural adaptation and validation

The process resulted in six Swedish versions of the CYP‐PREM, translated, culturally adapted, face‐ and content‐validated: inpatient and outpatient, for three age groups (8–11, 12–13 and 14–16 years), respectively. The number of questions in the final PREM varied between 25 and 34 (Table 3).

**Table 3:** Changes in the questionnaire after the cognitive interviews and CVI.

IP, OP	Original questions (*n*)	Questions changed (*n*)	Questions removed (*n*)	Question divided into two (*n*)	Questions removed after CVI testing (*n*)	Questions added to the final version (*n*)	Final version (*n*)
*IP 8–11*							
Total questions (*n*)	37	21	3	1	6		29
Hospital facilities	13	8	1	1	5		8
Treatment and tests	7	6					7
Hospital staff	7	3					7
After the visit	5	3	1		1		3
About you	5	1	1				4
*IP 12–13*							
Total questions (*n*)	44	24	4	1	9	1	33
Hospital facilities	16	10	1	1	6		10
Treatment and tests	8	6			1		7
Hospital staff	10	4	1		2	1	8
After the visit	5	3	1				4
About you	5	1	1				4
*IP 14–16*							
Total questions (*n*)	45	24	3	1	10	1	34
Hospital facilities	16	10	1	1	6		10
Treatment and tests	8	6					8
Hospital staff	11	4			3	1	9
After the visit	5	3	1		1		4
About you	5	1	1				4
*OP 8–11*							
Total questions (*n*)	30	17	3	1	3		25
Hospital facilities	5	4	1	1	3		2
Treatment and tests	7	7					7
Hospital staff	10	4					10
After the visit	3	1	1				2
About you	5	1	1				4
*OP 12–13*							
Total questions (*n*)	37	21	4	1	7		27
Hospital facilities	7	6	1	1	4		3
Treatment and tests	8	7					8
Hospital staff	14	6	1		3		10
After the visit	3	1	1				2
About you	5	1	1				4
*OP 14–16*							
Total questions (*n*)	38	21	3	1	7		29
Hospital facilities	7	6	1	1	4		3
Treatment and tests	8	7					8
Hospital staff	15	6			3		12
After the visit	3	1	1				2
About you	5	1	1				4

Abbreviations: CVI, content validity index; IP, inpatient; OP, outpatient.

In the cognitive interviews, the children experienced most questions as relevant and the layout of the questionnaire as easy to understand. Approximately half of the questions were modified as a result of the cognitive interviews; an overview of the questionnaire changes is provided in Table 3.

During the cultural adaptation process, several issues were identified regarding understanding of the questions in a Swedish context. An overview of the adaptations made is presented below and in Table 4.

**Table 4:** All issues identified and adapted in the various steps.

Issue no.	Pretesting Concept that could be unclear^a^ (why)	First interview round Concept not understood as intended^a^ (why)	Second interview round Concept not understood as intended^a^ (why)	Third interview round Concept not understood as intended^a^ (why)	Fourth interview round Concept not understood as intended^a^ (why)
	Which questionnaire(s)	Issue in which questionnaire(s)	Issue in which questionnaire(s)	Issue in which questionnaire(s)	Issue in which questionnaire(s)
	Adaptations made	Adaptations made	Adaptations made	Adaptations made	Adaptations made
1	The name of a specific children's hospital (1)	Issue resolved			
	IP				
	Changed to ‘the hospital’ and sometimes ‘the ward’ to be able to be used in all hospitals				
2	The name of a specific children's hospital (1)	‘The clinic/the outpatient ward’ (2, 3)	Issue remains	Issue resolved	
	OP	OP 8–11	OP 8–11 and OP 12–13		
	Changed to ‘the clinic/the outpatient ward’; allowed for the fact that outpatient clinics in Sweden can be situated outside the hospital	None. Further testing →	Omitted ‘the clinic/outpatient ward’, when possible to rephrase formulations, throughout the questionnaire. Slash replaced with ‘or’		
3	Language adjustments: Nurses, doctors, people looking after you, staff and so forth (4)	A few children were unsure of the meaning of the word ‘health care staff’ (2)	Issue remains	Issue remains	Issue resolved
	All	IP 8–11	OP 12–13	IP 8–11	
	Replaced with ‘health care staff’ for the questions to be uniform, both within and between the six versions of the questionnaire to make it easier to understand	None. Further testing →	None. Further testing →	Added information where the concept is used for the first time: ‘Health care staff are those who work in the ward, such as nurses and doctors’	
4	‘Column’ is a difficult word (2) that is commonly used in the instruction on answering the questions	Issue resolved			
	All				
	The word ‘column’ was omitted				
5	The names of the various rooms asked about (1)	Which room meant by ‘patient room’ (IP) and ‘treatment room’. Not clear which ‘waiting room’ intended (IP) (1)	Which room intended by ‘another room in the ward where you took samples or received treatment’ (1)	Issue resolved	
	All	All	All		
	Translated into ‘patient room’ (IP), ‘treatment room’, ‘consultant room (OP)’ and ‘waiting room’	Changed ‘patient room’ to ‘your room in the ward’ (IP) and ‘treatment room’ to ‘another room in the ward where they collected blood samples or you received treatment’. The description ‘waiting room’ was removed for IP	The description ‘treatment room’ was removed from the questions		
6	‘Adolescent room’ and ‘playroom’ were used in accordance with the respective age‐group (1, 4)	Issue resolved			
	All				
	‘Playroom’ and ‘adolescent room’ are added as response alternative in all versions of the questionnaire				
7	The name of a specific canteen at the specific children's hospital (1)	Had not been to ‘the cafeteria’. Unclear which cafeteria meant, and at which visit. (1)	Issue remains	Question removed	
	All	All	All		
	The name was replaced by ‘the cafeteria’	None. Further testing →	The question was removed		
8	Various examples of treatment and tests in the different versions (4)	The information text contains many difficult words (2)	Issue resolved		
	All	All			
	The examples were changed to make the information alike in all versions of the questionnaire	Deleted the text with examples and added sampling in the heading—‘Treatment, examination and sampling’, three overarching concepts			
9	‘If you were awake during your treatment or sample‐taking, did the nursing staff do any of the following to help you?’ (1)	Children misunderstood when reading the phrase ‘if you were awake’ (3)	The question was too extensive (3)	Issue resolved	
	All	All	All		
	The alternatives of reading a paper (including comics, papers and magazines) and listening to music were added as alternatives for distraction during procedures	Removed 'if you were awake’: ‘Did the health care staff do any of the following to assist you during your examination and treatment?’	Clarified that the question was about distraction. Removed response alternatives, keeping two alternatives: ‘Talked to you’ and ‘Other’, with information within brackets ‘(e.g., watching a movie or listening to music)’ Also added the ability to answer no		
10	Question about transition to ‘The adult hospital’ (1)	Not relevant yet (1)	Issue remains	Issue resolved	
	IP and OP, 12–13 and 14–16	IP and OP 12–13	IP and OP 12–13		
	Was changed to be about transition to ‘adult care’ as adult care could take place at the same health care setting	None. Further testing →	The question was removed for the age group 12–13 years		
11	Question about waiting for medicine before leaving the health care facilities is not applicable in the Swedish setting. Medicines are not picked up at the ward or outpatient clinic (1)	Medicines are not picked up at the ward or outpatient clinic (1)	Issue remains	Question removed	
	All	All	All		
	Testing	None. Further testing →	The question was removed		
12	Question about gender had two fixed alternatives: ‘Boy’ or ‘Girl’ (1)	No problem with the question	‘Own description’? (2)	Issue resolved	
	All		OP 8‐11		
	Alternatives other than boy and girl were added		The alternatives were kept		
13	Predefined first languages (1)	Issue resolved			
	All				
	The specified options of languages were removed, replaced by the option to fill in language as free text				
14	Postcode, uncertain whether question is relevant to the Swedish context (1)	The children did not know their postcode (1)	Issue remains	Question removed	
	All	All	IN 8–11, OP 8–11, OP 12–13		
	Testing	None. Further testing →	The question was removed		
15		‘How long did you have to wait… For each question put an X in a circle… for your tests, investigations or treatments?… to see a doctor or nurse’ (3)	Issue remains	Issue remains. Answer option for not remembering was also lacking (3)	Issue resolved
		OP 8–11	OP 12–13	OP 8–11	
		None. Further testing →	Simplified the layout of the question and adjusted the order according to what happens first at a visit ‘How long did you have to wait…… to see a doctor or nurse?’… for your tests, investigations or treatments? For each question, put an X in a circle	The question is divided into two questions. A response alternative ‘I don't know’ was added	
16		‘Activities’ difficult to understand (2)	Issue resolved		
		IP 8–11 and OP 8–11			
		Change the word for ‘activities’ to ‘things to do’			
17		Question about Wi‐Fi was difficult to understand, too extensive (3)	Issue remains Had only been in their room (1)	Issue resolved	
		IP 8–11, IP 12–13, OP 8–11	IP 8–11, IP 12–13, OP 12–13		
		None. Further testing →	Deletes the answer‐alternative ‘You could use it wherever you went in the hospital’		
18		As the children had not seen any other patients, the question about other patients was difficult to understand (3)	Issue resolved		
		IP 8–11, IP 14–16			
		Added response option: ‘I have not met any other children’			
19		‘ALL’—looks like abbreviation for acute lymphatic leukaemia (2)	Issue resolved		
		OP 8–11			
		Uses lowercase letters			
20		‘Did the health care staff prepare you before treatments or tests what was going to happen?’ (2, 3) Uncertain if the preparation was before or if it was about what would happen before the treatments or tests	Issue remains Also lacking response option about not needing any preparation due to previous visits	‘Before your treatments or tests, did the health care staff prepare you what was going to happen’ ‘Prepare’ was difficult to understand (2)	Issue resolved
		All	All	OP 8–11, OP 12–13	
		None. Further testing →	Clarified the question: ‘Before your treatments or tests, did the health care staff prepare you what was going to happen’ Added option ‘I have done this before, did not need any explanation’ in questions throughout the questionnaire	Changed to: ‘Before your treatments or tests, did the health care staff tell you what was going to happen’	
21		‘Did the health care staff spend enough time with you?’ ‘Spend’ and ‘enough’ are difficult to understand (2)	Issue remains	Issue resolved	
		OP 8–11, OP 12–13, IP 8–11	IP 8–11		
		None. Further testing →	Simplified wording ‘Were the health care staff with you for the time you wanted?’		
22		‘Did the health care staff give you information about why you were in the ward?’ ‘Information’ difficult to understand (2)	Issue remains	Issue resolved	
		OP 8–11	OP 8–11		
		None. Further testing →	Simplified word choice. ‘Did the health care staff tell you why you were in the ward?’		
23		‘Did the health care staff encourage you to ask questions if you were wondering anything during your visit?’ ‘Encourage’ difficult to understand (2)	Issue remains	Issue resolved	
		All	All		
		None. Further testing →	Simplified word choice. ‘Did the health care staff ask you if there was anything you were wondering about during your visit?’		
24		‘Do you feel that you had a say in any decisions that were made about your care?’ difficult to understand (1, 2)	Issue remains	Issue resolved	
		OP 8–11	IP 8–11, OP 8–11, OP 12–13		
		None. Further testing →	Simplified the question. ‘Did you get to be involved and decide on your care?’		
25			The translation ‘admitted to hospital’ was difficult to understand (1)	Issue resolved	
			IP 8–11		
			Was replaced with ‘slept at the ward’		
26			‘Did you meet with a doctor or nurse alone?’ Difficult to know what profession of doctors they had met (3, 4)	Issue resolved	
			All		
			Changed to ‘health care staff’ as in other questions		
27				‘How would you describe your experience of your visit to the hospital?’ Complicated sentence (3)	Issue resolved
				OP 8–11	
				Simplifies the question: ‘How did you experience…’	

Abbreviations: All, inpatient and outpatient all ages; IP, inpatient all ages; IP 8–11, inpatient 8–11 years; IP 12–13, inpatient 12–13 years; IP 14–16, inpatient 14–16 years; OP, outpatient all ages; OP 8–11, outpatient 8–11 years; OP 12–13, outpatient 12–13 years; OP 14–16, outpatient 14–16 years; →, further testing.

^a^ Reason for change: 1 = *Not relevant or difficulty understanding due to context*, 2 = *Difficulty understanding the meaning of the words*, 3 = *Difficulties in understanding the question's structure or layout*, 4 = *Inconsistent terminology*.

#### 5.1.1 Not relevant or difficulty understanding due to context

Some cultural adaptations were needed early in the translation process throughout all versions of the questionnaire to make them useful in a Swedish context. The questionnaire, which was originally designed for use in a specific children's hospital in the United Kingdom, was adapted to be used in a Swedish health care context regardless of the hospital or outpatient clinic. For example, the name of the UK hospital was replaced with the Swedish words for ‘the hospital’ or ‘ward’ in the inpatient versions and ‘the clinic/the day‐care‐ward’ in the outpatient versions.

Gender alternatives other than boy and girl were added to the demographic section. The question about children's first language was changed to be open‐ended due to the difficulty of including all relevant languages. These changes were made in the pretesting phase so that everyone answering the questionnaire would feel included.

Three questions were removed after the second round of cognitive interviews due to contextual problems. For example, cafeterias in Swedish hospitals are often run by external companies and it was noted that children rarely visited cafeterias in conjunction with the health care visit. Another example of a contextual problem concerned waiting for medicines before leaving the hospital, which was not relevant because in Sweden, all medicines are obtained with a prescription from a pharmacy separately from the clinic. The children also did not know their postcode and this question was not considered relevant to the Swedish context and was therefore removed.

In questions about hospital facilities, it was challenging to find the appropriate name for the rooms that children may have been in during their visit (Table 5). Before testing, names of rooms were adapted to the Swedish context, but children still found this difficult to understand. After the first round, ‘treatment room’ was changed to ‘another room in the ward where the health care staff collected samples, or you received treatment’. However, it was still not clear which room was being referred to and many children received their treatment in the consultation room in the clinic/outpatient ward or their room on the ward. Questions about treatment rooms were therefore removed after the second round.

**Table 5:** Examples of children's views of the questionnaire.

Questions about the environment in different rooms	Question about distraction	The extent of the questionnaire	The layout of the questionnaire	Question about male or female health care staff
Patient room Waiting room Playroom/youth room Treatment room	If you were awake during your treatment or test, did the health care staff do any of these things to help you?	The number of questions in the original versions of CYP‐PREM vary between 31 and 45	Texts about animals and illustrations (8–11 and 12–13 years) Cartoons (14–16 years)	Did you have the opportunity to choose whether you wanted to meet male or female health care staff?
Difficulties in identifying different rooms *‘*Patient room, hhmm this was hard to know’. (Boy, 9 years) ‘Patient room, is that where I was examined?’ (Girl, 12 years) ‘Patient room, is that this room or?’ (Boy, 16 years) ‘Treatment room? I do not understand that’. (Girl, 14 years) ‘I do not really know what kind of room this is, it feels like a treatment room because I have received treatment in it’. (Girl, 11 years)	Children misunderstood when reading the phrase ‘if you were awake’ *‘*It's hard because you were not awake, it still says awake, it can be a little hard to remember’. (Girl, 10 years) ‘I've never been awake, so I'm just ticking this—they did not do any of this’ (has an ongoing treatment in a newly inserted intravenous catheter). (Boy, 11 years)	Many of the younger children said that the questionnaire was too comprehensive ‘Whew, many pages … It feels like I am doing homework’. (Girl, 10 years) *‘*How many pages are there …?’ (Girl, 10 years) ‘There are 44 questions! It can take a lot of time’. (Girl, 12 years [do not complete the questionnaire]) ‘This will take me an hour (sigh)’. (Boy, 8 years) ‘Finally, the last page!’. (Boy 12 years)	About the animal texts: ‘It is very fun with facts to learn new things about animals’. (Girl, 11 years) ‘Feels pretty fun, but I will not remember all the facts’. (Boy 12 years) About the animal illustrations: ‘If you are younger, you may think it's fun, it does not matter to me’. (Girl, 13 years) About the cartoons: ‘The older you get, the more boring forms like this become. It makes it easier to concentrate’. (Girl, 15 years) ‘It describes different people … shows that everyone feels different, it should be better for everyone’. (Boy, 15 years)	Individual children would have preferred to choose, but most expressed that it was not important to them, but could be important to others ‘Did not get the question, a man helped me to the toilet, had felt better with a female, depends on what to do’. (Girl, 14 years) ‘I have not been given the opportunity to choose. I have had both female and male health care staff and I do not think any of it was unpleasant, so for me it doesn't matter’. (Girl, 13 years) ‘For some it may be important, for me it does not matter at all’. (Boy, 15 years)

Abbreviation: CYP‐PREM, Children's and Young People's Patient‐Reported Experience Measure.

#### 5.1.2 Difficulties understanding the meaning of the words

Linguistic adaptations were made when children did not know/understand the meaning of words and most difficulties were identified in the first round. Some words were directly replaced, for example, text about treatment and tests that included many difficult words (such as injections, cannulas, catheters) was simplified. However, some words were tested in a further round. In most cases, synonyms were used when making changes. The words ‘information’ and ‘health care staff’ were difficult for the youngest children to understand but were considered difficult to replace. The word ‘information’ was replaced in one question: ‘Did the health care staff *tell you* why you were in the ward?’ but kept in another: ‘Was the *information* they gave you about why you were in hospital easy to understand?’. Concerning ‘health care staff’, further detail was added in all versions of the questionnaire to clarify the meaning of the concept the first time it was mentioned: ‘Health care staff are those who work in the ward, such as nurses and doctors’.

#### 5.1.3 Difficulties in understanding the question structure or layout

Problems understanding the question arose in some cases due to the layout of the question or a lack of response options that seemed appropriate to the children. Adjustments were made to make it easier to understand how the questions should be answered. For example, children lacked a response option for situations when information about a procedure was not given because of the child's previous experiences. The response option ‘I have done this before, did not need any explanation’ was added. Another example is a question that was divided into two: ‘How long did you have to wait … to see a doctor or nurse?’… for your tests, investigations or treatments? Some children got stuck on the question and did not know how to answer. The question was split into two questions to resolve the problem.

#### 5.1.4 Inconsistent terminology

Textual changes were made consistent in all versions. For example, ‘playroom’ and ‘adolescent room’ were added as response options in all versions, because of the possibility of younger children using adolescent rooms and teenagers spending time in playrooms.

Sometimes, children had difficulty understanding which visit questions related to; they often talked about other hospital visits and answered the questions in relation to this. The final Swedish version will include information explaining that the questionnaire concerns the most recent visit with space left for the investigating clinic/hospital to add their details.

Most children were happy with the graphic design of the questionnaire, although individual children in the older groups said that the design did not matter to them but it could be helpful for younger children (Table 5). Many younger children said that the questionnaire was too extensive but could not say that any question should be removed.

### 5.2 Content validity

Questions that received high CVI (were regarded as most important) in all age groups mostly concerned the staff. Examples are questions about feeling welcome, being treated with respect, being listened to and feeling trust. Questions about the food and the opportunity for privacy were also highly valued. The questions rated the lowest were room temperature, age of other patients on the ward and whether the children were offered the choice to see male or female staff. The number of questions with I‐CVI 0.57 or lower varied between 4 and 10 for the six versions of CYP‐PREM (Table 6), giving these versions of the questionnaire an S‐CVI/Ave between 0.70 and 0.82. Questions graded ‘not so important’ or ‘not at all important’ by three or more of the seven participating children were excluded. However, an open free‐text question with the opportunity to express what was good and bad at the health care visit was rated low by the participants but was nevertheless considered important by the research group and therefore not excluded. This also applied to questions about participation in decisions about care and the possibility of meeting staff without parents/carers. The research group decided that the question about the possibility of ‘meeting staff on your own’ should in fact be included in all versions of the questionnaire for children from the age of 12, which led to the question being added to two versions of the questionnaire for inpatient care. The question was added after the CVI was conducted and has thus not been evaluated by inpatient children in the age groups 12–13 and 14–16 years. This question is, therefore not included in the final CVI calculation for these versions of the questionnaire. In the final versions, 3–10 questions have been excluded from the different versions of the CYP‐PREM (Table 3), leading to an improvement of relevance and the S‐CVI/Ave, which now varies between 0.81 and 0.94 for the six versions of the questionnaire (Table 6).

**Table 6:** CVI results.

Version	S‐CVI in tested version	Content of questions with CVI fair (0.57) or lower, indicating removal	Question added	S‐CVI in new version
IP 8–11	0.79	Wait for staff 0.57 Wait for bed 0.29 About the rooms 0.57 Things to do 0.57 Age of other patients 0.14 Wait to go home 0.29		0.89
IP 12–13	0.79	Wait for bed 0.57 Room temperature 0.43 Things to do 0.43 WiFi 0.57 Own room 0.57 Age of other patients 0.14 Distraction during procedure 0.57 Choose male or female staff 0.29 Info about why in the ward 0.57	Seen staff on your own^a^	0.90
IP 14–16	0.82	Wait for bed 0.57 Noise 0.43 Room temperature 0.29 About things to do 0.57 WiFi 0.57 Age of other patients 0.29 Choose male or female staff 0.43 Information about transition to adult care 0.57 Information about adult care 0.57 Wait to go home 0.57	Seen staff on your own^a^	0.94
OP 8–11	0.80	About the rooms 0.43 Wait for staff 0.57 WiFi 0.43 Good or bad about the outpatient visit 0.57^b^		0.84
OP 12–13	0.70	Noise 0.43 Space 0.43 Room temperature 0.14 WiFi 0.29 Choose male or female staff 0.57 Eye contact during conversations 0.29 Info about why in the ward 0.43 Seen staff on your own 0.14^b^		0.81
OP 14–16	0.77	Noise 0.43 Space 0.57 Room temperature 0.43 Things to do 0.57 Choose male or female staff 0.43 Good or bad about the outpatient visit 0.57^b^ Eye contact during conversations 0.57 Have a say in decisions 0.57^b^ Information about transition to adult care 0.43 Seen staff on your own 0.29^b^		0.85

Abbreviations: CVI, content validity index; IP, inpatient; OP, outpatient; S‐CVI, scale‐CVI.

^a^ The added question is not included in the S‐CVI/Ave and thereby not evaluated by the children.

^b^ Questions kept after discussion within the research group.

To summarise, the translation, cultural adaptation and validation resulted in six face‐ and content‐validated Swedish versions of the questionnaire ready for pilot testing.

## 6 DISCUSSION

In view of the lack of a Swedish nationally validated PREM for completion by children, our aim was to translate, adapt and validate the six versions of the CYP‐PREM questionnaire^13^ for use in a Swedish health care context. The adaptation and validation process^17^ identified several issues regarding understanding of the questionnaire in the new setting, and the main finding of this study is confirmation of the importance of a rigorous validation. Cognitive interviews are considered valuable for pretesting questions for specific groups for whom it may be particularly difficult to complete questionnaires,^20^, ^21^ and were therefore used with children in this study. Adaptations were made based on issues related to context, words used and the structure of the questions and to obtain a consistent terminology. Many adaptations are similar to those made in the Danish versions of CYP‐PREM, for example, regarding different rooms.^14^ The cognitive interviews showed that the questionnaire was too comprehensive, but which questions were least relevant to the participants could not be determined. Addition of CVI^18^ and the development of the study design to an exploratory sequential mixed‐method design^16^ have increased and clarified the relevance of the questionnaire in the Swedish health care context.

### 6.1 Strength and limitations of the work

Translation and validation of an existing questionnaire have both advantages and disadvantages. A major advantage of adapting the CYP‐PREM^13^ to the Swedish health care context was that the questionnaire evaluates issues known to be important to children: competence of health care staff, opportunity for participation, adapted communication and environment, presence of parents/carers, things to do and hospital food,^27^, ^28^ and also has a layout that appeals to children.^14^

However, although the CYP‐PREM was developed in collaboration with children in the United Kingdom, the questionnaire could not be used with children in another health care context without appropriate cultural and linguistic adaptation, which Ryberg et al.^14^ also demonstrated in their adaptation of CYP‐PREM to Danish outpatient care. It is a strength of the present study that children who have experience in what is being studied are involved, the importance of which has been highlighted by the Council of Europe.^6^, ^29^

Translation, adaption and validation of the questionnaire have been extensive and demanding tasks, partly since there were six different versions to handle simultaneously. It was also a challenge to formulate the questions to be suitable for all children who come into contact with health care, regardless of which health care facility the visit concerns and the reason for the health care visit. It was considered sufficient to have eight children in round four and no inpatients aged 14–16 years, since few changes had been made and it was more important to test understanding in the youngest groups. One strength of the study is that children of different ages, with different first languages, illnesses and experiences from different health care facilities, were involved in the validation process. Besides the strength of including the perspectives of children with a range of demographic characteristics, it was also regarded as a strength that the interviewers had professional knowledge and understanding of children and paediatric health care.^11^ Furthermore, from an ethical perspective, it was important that the researchers were experienced in talking with, and listening to, children of different ages, but on the other hand, it was also challenging to ask sufficient follow‐up questions to fully explore children's understanding without pushing them too much.^30^ In research with children, the influence of adults cannot be completely excluded. Children can feel both pressured and/or prevented from participating, so‐called gate‐keeping,^26^ and adults can also try to influence the children's responses, which, in this study, could have happened during the data collection for the CVI, which was carried out without the presence of a representative from the research group. However, due to the homogeneity in children's rating of relevant, and less relevant, questions in the CYP‐PREM, we regard this risk as low.

Exclusion of questions was based on children's views by calculation of I‐CVI in the six different versions of the CYP‐PREM, although some questions rated low by the children have been retained since they were considered important to the health care departments in evaluation of how the children's rights are respected. The assessment of relevance in the current study was a balancing act between the children's opinions and the professional perspective about children based on laws and guidelines.^4^, ^5^, ^6^ For example, a question about having the opportunity to be involved in decisions on care and an open question that gives the respondent the opportunity to express points of view that might otherwise be missed were deemed important from a children's rights perspective.^5^, ^6^ Also retained in the CYP‐PREM was a question about being given the opportunity to meet staff on their own, as it was assessed by the research group to be important that it was included in all versions of the questionnaire for children from the age of 12 years. This led to the question being added to the versions for inpatients 12–13 and 14–16 years (it was already in the outpatient versions for these ages). The added question was not included in the calculation of S‐CVI/Ave.

To the best of our knowledge, only a few translation, adaptation and validation studies of PREMs for children have been undertaken previously. This study confirms the importance of including children themselves so that questionnaires are developed based on their views.^31^ Issues arising in the cognitive interviews were not all predicted despite the research group's experience of paediatric health care, which further emphasises the need for collaboration with the target group. A strength of this study is that an established method to adapt the questionnaire has been followed^17^, ^18^, ^19^ and outlined in detail, which enables other researchers to replicate the process.

### 6.2 Recommendations for future research

Further pilot testing and evaluation of the psychometric properties of the adapted CYP‐PREM are needed to assess the acceptability and feasibility of using the questionnaire to evaluate experiences of paediatric health care in Sweden. Further research is also needed regarding the implementation of PREMs in paediatric health care and to investigate if the CYP‐PREM can be used in other healthcare contexts, for example, with children in psychiatric care. A questionnaire adapted for younger children and children with learning disabilities is needed in order to allow additional groups of children to have equal opportunities to voice their experiences about their care, and this is currently being undertaken by the UK team.

### 6.3 Implications for policy and practice

The face‐ and content‐validated versions of the CYP‐PREM are relevant in the Swedish health care context and can increase the opportunities to enable children to voice their experience of health care in paediatric wards in Sweden. The importance for children to feel involved is a prerequisite for them to achieve autonomy. Therefore, it is necessary that the children are allowed to indicate which factors they think are important in order to feel that their participation is valued and heard and to rank what is important to them. This study has highlighted the importance of ensuring that questionnaires are linguistically and culturally adapted in collaboration with representatives of the population with whom they will be used. The steps used in the translation, cultural adaptation and validation process can be recommended for other similar studies, thereby enabling children from other cultural and linguistic backgrounds to voice their experiences of health care, which is not only a fundamental right but also vital to ensure that services evolve to meet their needs. The culturally adapted Swedish versions of CYP‐PREM will be available to health care professionals so that they can monitor the quality of children's experiences of healthcare visits. In addition, the questionnaire will enable comparisons between paediatric units in Sweden.

## 7 CONCLUSION

Although the original CYP‐PREMs were developed in collaboration with children in the United Kingdom, this does not mean that they can be automatically used with children in a Swedish health care context. To ensure quality and applicability of the PREMs to children accessing health care in different countries, a rigorous process of translation and cultural adaptation is important, which this study illustrates. The interviews identified the need for adjustments regarding context, wording, question structure and layout. The CVI testing increased and clarified the relevance of the questions in the Swedish health care context and led to a questionnaire with fewer questions, which can be regarded as more user‐friendly.

The six Swedish versions of the generic CYP‐PREM are now deemed to be understandable to, and relevant for use by, children in a Swedish health care context.

## AUTHOR CONTRIBUTIONS

All authors contributed to designing the study, in the analysis, with intellectual content, approved the final version of the manuscript and agreed to be accountable for all aspects of the work. Anna Nordlind and Ann‐Charlotte Almblad in addition conducted the cognitive interviews, fed the data into a case‐specific matrix and initiated the analysis of the cognitive interviews. Anna Nordlind and Ann‐Charlotte Almblad collected data for content validation index (CVI), Anna Nordlind and Ann‐Sofie Sundqvist summarised CVI data, Anna Nordlind calculated CVI and all authors together decided where the limit for CVI should be set and which questions needed to be excluded. Anna Nordlind was responsible for writing the manuscript. Jo Wray and Geralyn Oldham have reviewed the language in the manuscript.

## CONFLICT OF INTEREST STATEMENT

The authors declare no conflict of interest.

## ETHICS STATEMENT

The study was approved by the Swedish Ethical Review Authority (Dnr 2019‐01203, 2020‐02350, 2022‐00837‐02).

## Supporting information

Supporting information.

Supporting information.

Supporting information.

Supporting information.

## Data Availability

The data that support the findings of this study are available on request from the corresponding author. The data are not publicly available due to privacy or ethical restrictions.
