# Supplementary figures and images for: Translation, cultural adaptation and validation of a patient‐reported experience measure for children
Source: Health Expect. 2023 Dec 7;27(1):e13924. doi: 10.1111/hex.13924 (PMC10768864; doi:10.1111/hex.13924)

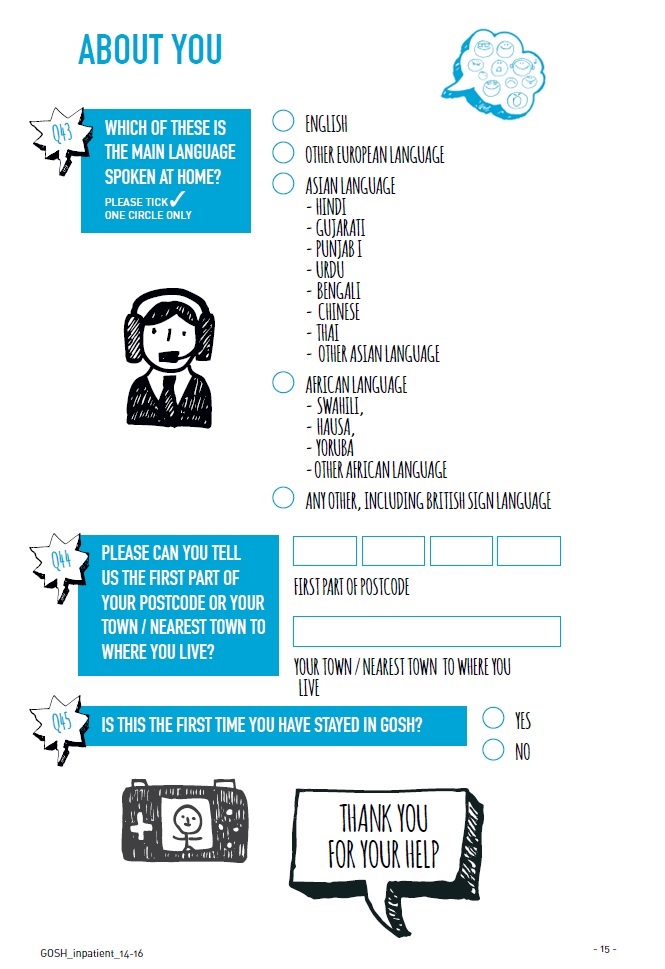

Supplement: Supplementary file 1 — Supporting information. [file HEX-27-e13924-s004.jpg]

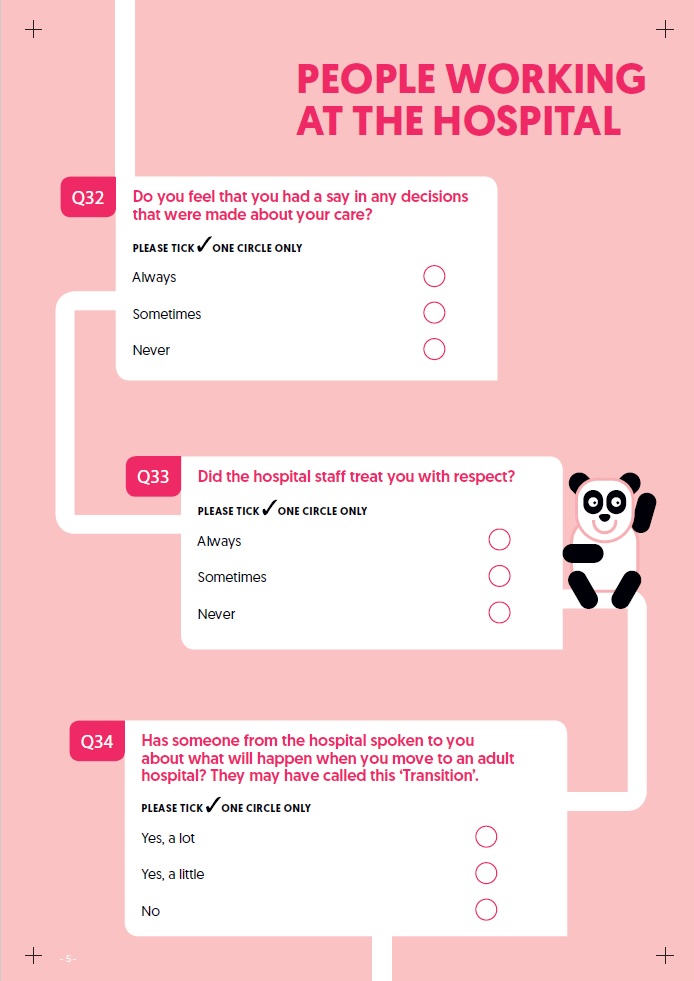

Supplement: Supplementary file 2 — Supporting information. [file HEX-27-e13924-s002.jpg]

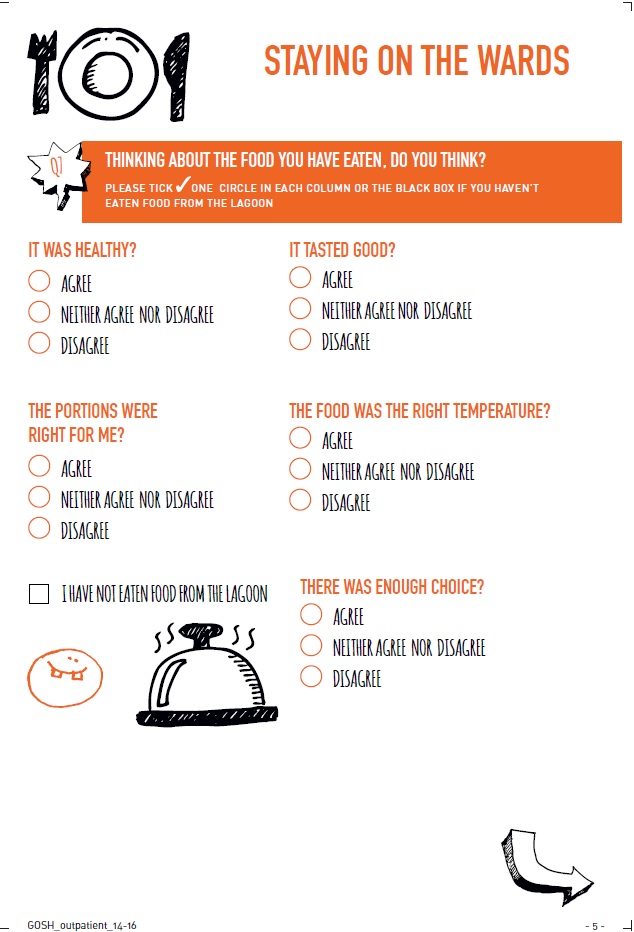

Supplement: Supplementary file 3 — Supporting information. [file HEX-27-e13924-s003.jpg]

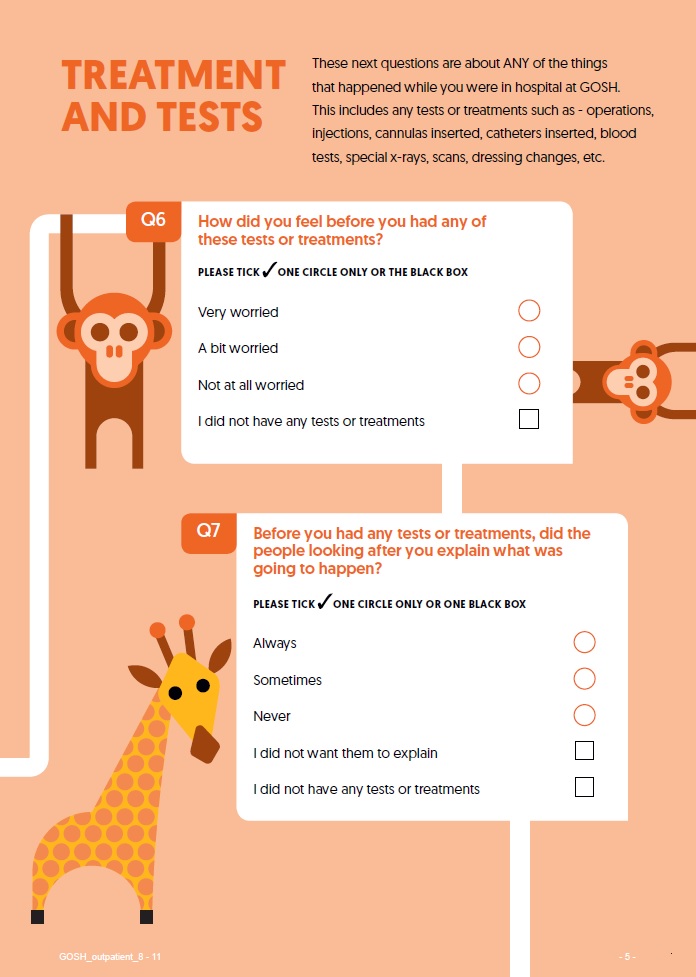

Supplement: Supplementary file 4 — Supporting information. [file HEX-27-e13924-s001.jpg]
